# Ultra-rare complement factor 8 coding variants in families with age-related macular degeneration

**DOI:** 10.1016/j.isci.2023.106417

**Published:** 2023-04-03

**Authors:** Lina Zelinger, Tammy M. Martin, Jayshree Advani, Laura Campello, Milton A. English, Alan Kwong, Claire Weber, Jennifer Maykoski, Yuri V. Sergeev, Robert Fariss, Emily Y. Chew, Michael L. Klein, Anand Swaroop

**Affiliations:** 1 Neurobiology, Neurodegeneration and Repair Laboratory, National Eye Institute, National Institutes of Health, Bethesda, MD, USA; 2 Casey Eye Institute, Department of Ophthalmology, Oregon Health & Science University, Portland, OR, USA; 3 Department of Molecular Microbiology & Immunology, Oregon Health & Science University, Portland, OR, USA; 4 Department of Biostatistics and Center for Statistical Genetics, University of Michigan, Ann Arbor, MI, USA; 5 Division of Epidemiology and Clinical Applications, Clinical Trials Branch, National Eye Institute, National Institutes of Health, Bethesda, MD, USA; 6 Ophthalmic Genetics and Visual Function Branch, National Eye Institute, National Institutes of Health, Bethesda, MD, USA; 7 Biological Imaging Core, National Eye Institute, National Institutes of Health, Bethesda, MD, USA; 8 23andMe, Inc, Sunnyvale, CA, USA

**Keywords:** Genetics, Molecular biology, Molecular Genetics, Molecular Structure, Molecular interaction

## Abstract

Genome-wide association studies have uncovered 52 independent common and rare variants across 34 genetic loci, which influence susceptibility to age related macular degeneration (AMD). Of the 5 AMD-associated complement genes, complement factor H (CFH) and CFI exhibit a significant rare variant burden implicating a major contribution of the complement pathway to disease pathology. However, the efforts for developing AMD therapy have been challenging as of yet. Here, we report the identification of ultra-rare variants in complement factors 8A and 8B, two components of the terminal complement membrane attack complex (MAC), by whole exome sequencing of a cohort of AMD families. The identified C8 variants impact local interactions among proteins of C8 triplex *in vitro*, indicating their effect on MAC stability. Our results suggest that MAC, and not the early steps of the complement pathway, might be a more effective target for designing treatments for AMD.

## Highlights

• Ultra-rare C8A and C8B coding variants in age-related macular degeneration families
• C8 variants may also contribute to genetic risk in non-familial AMD cases
• C8 variants affect subunit interactions and may impact MAC stability

## Graphical abstract

**Figure:**
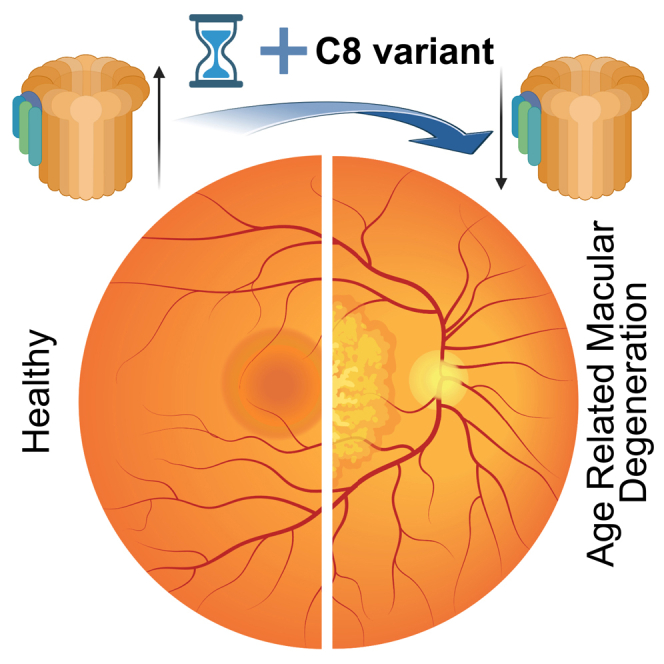

## Introduction

Age-related macular degeneration (AMD) is a late-onset neurodegenerative disease, which is a major cause of vision impairment in elderly population and constitutes 6–9% of legal blindness globally.^1^ Prevalence of AMD is estimated to be over 280 million within the next 20 years^2^ presenting a significant socioeconomic burden on individuals and society at large. Advanced age, environmental factors, lifestyle, and genetic predisposition contribute significantly to disease pathologies, which include disruption of retinal pigment epithelium (RPE) function, formation of large drusen and progressive degeneration of macular and perimacular photoreceptors.^3^^,^^4^^,^^5^ Advanced AMD is classified as wet (because of subretinal neovascularization, termed neovascular AMD or nAMD) or dry (regional geographic atrophy (GA) with no neovascularization).^1^ Early familial clustering and linkage studies suggested multiple genomic regions of potential AMD susceptibility.^5^ Later, a large genome-wide association study (GWAS) uncovered 52 common and rare variants at 34 genomic loci that help explain over half of the AMD heritability,^6^ with the complement system emerging as a major contributor,^5^^,^^7^^,^^8^ together with chronic inflammation and disruption of the extracellular matrix. However, pathophysiological mechanisms of AMD progression remain unclear hindering effective diagnosis and development of therapies.

Population and GWAS-based strategies have been widely successful in identifying risk factors for multiple neurological conditions, but for the most part these risk alleles are present in non-coding or inter-genic parts of the genome making the interpretation of their function and association to specific genes or molecular mechanisms extremely difficult. In search of significant missing heritability and rare variants to target specific genes, we previously analyzed families with advanced AMD and suggested association in 13 additional genes.^9^ In this study we leverage the advantages of familial analysis to identify ultra-rare variants in complement Factor 8A (*C8A*) and 8B (*C8B*) genes, which segregate with advanced AMD in 4 unrelated families. Complement factor 8 is part of the terminal step of the complement cascade, forming the membrane attack complex (MAC). Our results allow for a better understanding of the genetic factors contributing to disease development and propose a plausible and unifying model of MAC-associated AMD pathology.

## Results

### Genetic analysis identifies complement factor 8 variants in a familial cohort

**Figure 1:**
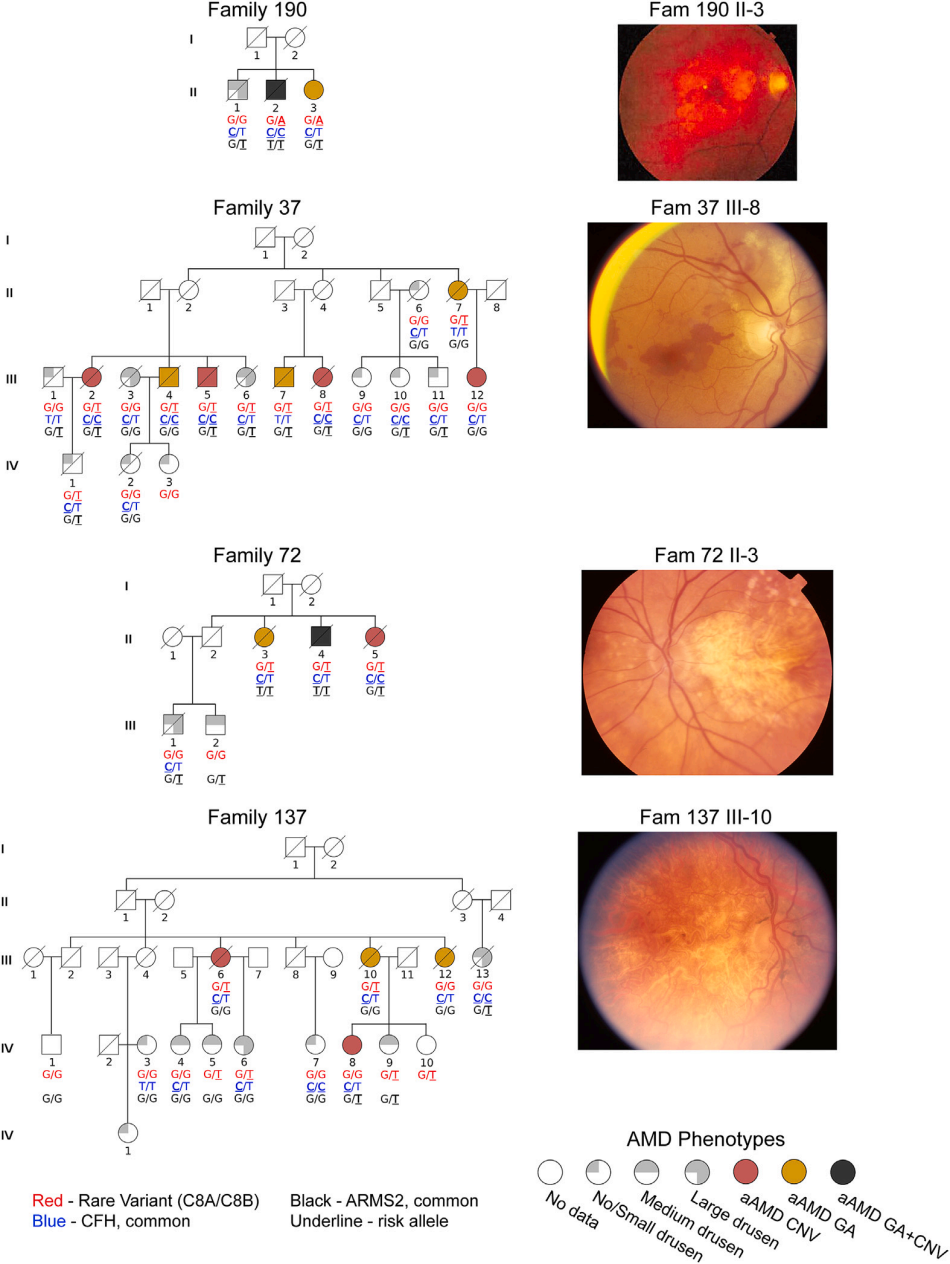
Rare C8 variants segregate in familial AMD cases Pedigree structures of four AMD families, with symbols indicating sex and disease phenotype, and genotypes of rare *C8A* or *C8B* variants as well as two common large effect AMD risk alleles noted below each symbol (see keys at bottom of the figure). To the right of each pedigree, fundoscopy images of select individuals with advanced AMD are shown. aAMD - advanced age-related macular degeneration; GA - geographic atrophy; nAMD-neovascular age-related macular degeneration.

**Figure 2:**
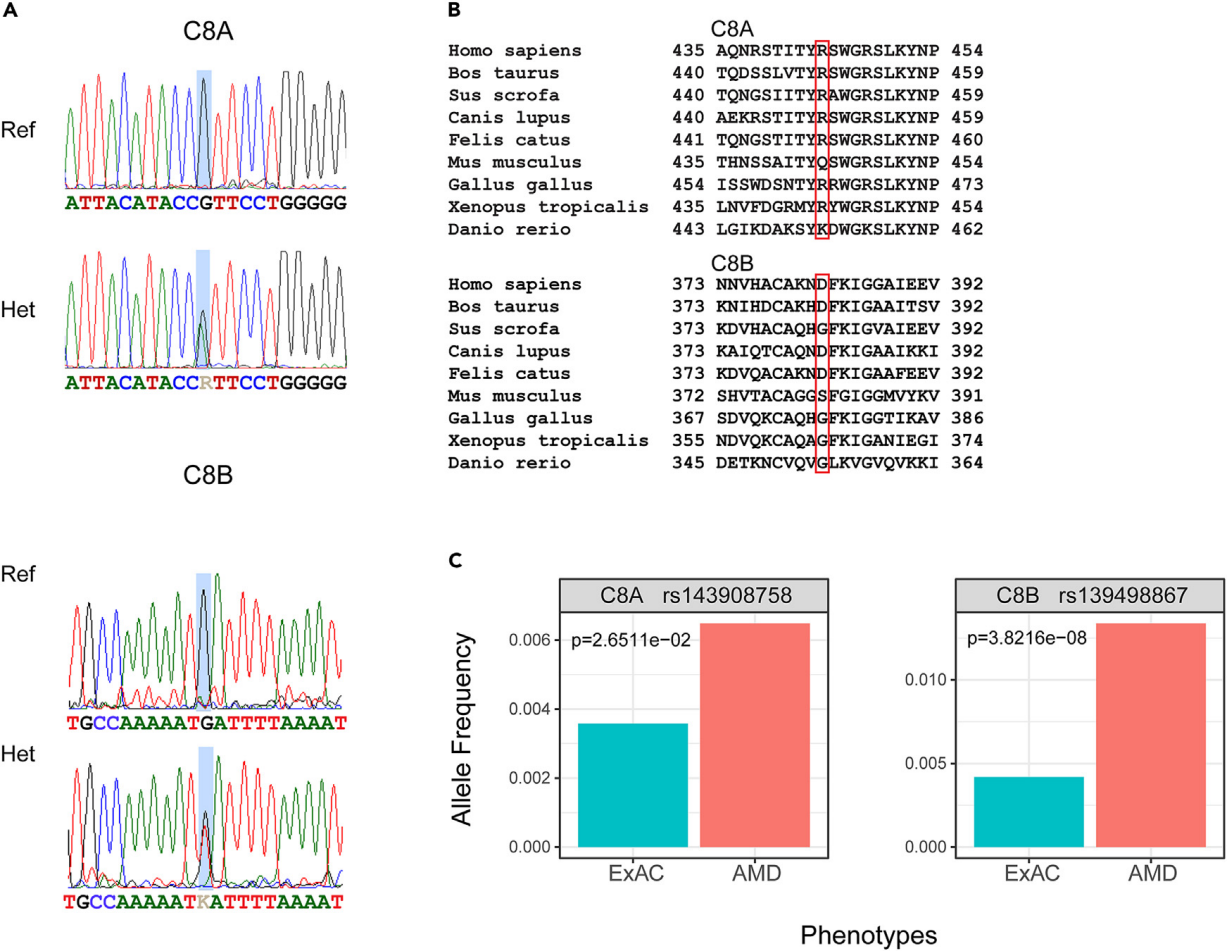
Conservation of C8 variants and carrier frequency (A) Sequence chromatograms of C8A and C8B variants from affected (carriers) and healthy individuals. The position of the variant is highlighted by a light blue rectangle and sequence is indicated under each chromatogram. (B) Protein level conservation of residues in C8 subunits. Location of the variants is indicated by a red rectangle. (C) Carrier frequencies of C8 variants in AMD affected individuals from the AREDS 1 & 2 cohorts compared to publicly available carrier frequencies in the general population. P-values are indicated for each variant.

Four families with multiple members diagnosed with advanced AMD (multigenerational manifestation of AMD in 3 families) were recruited for the study (Figure 1, See also Data S1-Table S1). A modified AREDS phenotype was assigned to 37 participants based on fundus photographs and relevant medical history (see STAR Methods for details) out of 39 enrolled in the study (age 52-89 years; Data S1 - Table S1). Diagnosis of advanced AMD was made when either GA, nAMD, or both were present in at least one eye. Inheritance patterns within the families suggest an autosomal dominant mode of inheritance, indicating a genetic variant with a large effect, which prompted us to perform whole exome sequencing (WES) on 13 affected and 4 unaffected individuals from these families to identify potential disease-associated variant/s. The initial screen of all previously reported AMD genes revealed no rare or pathogenic segregating variants. Further analysis uncovered two putative causal variants (See also Table S2); one in *C8A* (c.G1331A, p.R444H, ClinVar: rs143908758 in one family), and the other in *C8B* (c.G1144T, p.D382Y, ClinVar: rs139498867, in three families). We validated WES findings by performing Sanger sequencing of 21 additional family members (Figure 2). The identified missense variants are observed at extremely low frequencies in public databases (Global MAFs of 0.00140 and 0.00280, respectively, https://www.ncbi.nlm.nih.gov/clinvar/), are predicted to be pathogenic by SIFT, PolyPhen, and CADD (See also Table S2, and Figure S1) and alter either conserved or semi-conserved residues in the protein (Figure 2).

Two common AMD risk variants are known to have a large genetic effect (ClinVar: rs1061170 in *CFH* and ClinVar: rs10490924 in *ARMS2*), but neither segregated well with the disease in the four families (Figure 1). In Family 37, individual II-7 did not carry either of the common risk variants. Notably, affected individuals homozygous for the *CFH* rs1061170 risk allele in addition to a rare C8 variant were more likely to develop neovascularization. This co-occurrence was observed in five out of the six CFH homozygous patients, corresponding to a rate of 83% (4/6 nAMD versus 1/6 GA versus 1/6 both nAMD+GA; See also Table S1). Although the low number of individuals does not allow for statistical analysis, these data suggest that a cumulative burden in the complement cascade can drive nAMD development, consistent with the previously proposed multi-hit hypothesis.^5^

### C8 variants are prevalent in AREDS1 and AREDS2 cohorts

To investigate the role of C8 variants on a population level, we interrogated the available sequencing data from the two large AMD cohorts that participated in the AREDS 1 and 2 studies.^10^^,^^11^ Our analysis of the genotyping data from AREDS1/2 AMD cohorts revealed that while the overall frequencies of these variants remained low, these were significantly higher compared to those reported in public databases (Figure 2). Our data indicate that these ultra-rare C8 variants could be contributing to genetic risk even in non-familial AMD cases.

### Complement factor 8 is part of the membrane attack complex and is expressed by the RPE

**Figure 3:**
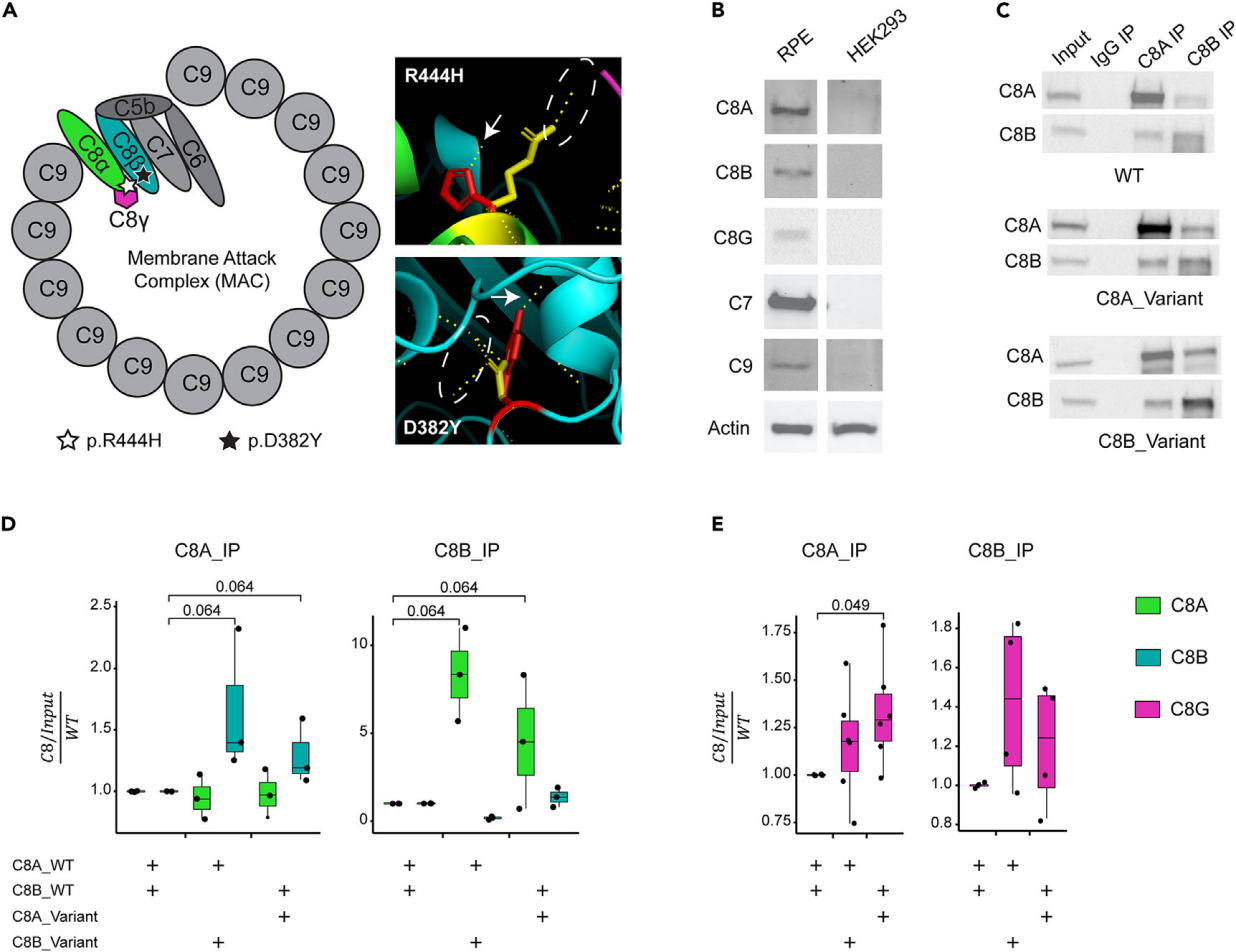
Pathogenicity and effect of variants on protein function (A) Representation of the structure of MAC and C8 complex. The schematic representation of MAC shows the location of the identified variants in protein complex. 3D visualization of C8 complex showing the three subunits (C8α – green, C8β – teal, C8γ – magenta). Variants are indicated by red residues, and WT residues are in yellow. Potential hydrogen bonds formed by residues are indicated by dashed yellow lines. Hydrogen bonds gained by introduction of variants are marked by white arrows, and hydrogen bonds lost are marked by a dashed white ellipsoid. (B) Immunoblots showing expression of C8 and its direct partners within the membrane attack complex (MAC) in human retinal pigment epithelium (RPE) and lack of expression in HEK293 cells. (C) Co-IP analyses on the interaction between C8 α, β, and γ subunits in protein lysates from HEK293 cells. (D and E) Quantification of IP signal in lysates from HEK293 cells. Relative amounts of C8 subunits are plotted on a Jitter plot. Individual measurements are marked by a dot, and the quadrant range is indicated by a box. Transfection (repeated in triplicates) conditions are indicated by a “+” under each panel. Quantification of C8 α, β, and γ subunits is indicated in green, teal and magenta, respectively. Wilcoxon test was done to assess statistical significance, and comparison approaching significance are indicated.

C8 is a protein triplex comprising two main subunits (α and β), and a smaller γ subunit which is not essential for MAC formation. The variants we identified are located in the main subunits and could impact both interactions with C8γ as well as other components of the MAC (Figure 3). There is an ongoing debate as to the primary location/cells that initiate the disease process; nonetheless, it is well established that RPE function is significantly affected in AMD. Therefore, we first confirmed the presence of C8 subunits, with C7 and C9 (known interacting partners in the formation of the membrane attack complex) in human donor RPE, a key target cell type in AMD (Figure 3).

### In-silico analysis and modeling predict that C8 variants affect protein interaction

We then constructed a homology model of the C8 heterotrimeric complex (STAR Methods, Data S2) and introduced the variants in the α and β subunits to evaluate their potential impact on local interactions (Figure 3; See also Figure S2). R444H variant in C8α is predicted to result in the addition of a hydrogen bond that would interact with a residue on C8β (Figure 3) and perturb another residue reported to interact with C8γ (PDB: 3OJY, https://www.ebi.ac.uk/pdbe/entry/pdb/3ojy/protein/1). D382Y variant in C8β is anticipated to disrupt local structure with the loss and addition of several H-bonds (Figure 3). Protein stability calculations predicted that both variants have non-native stable conformations (See also Table S3), with C8β D382Y indicating a more pronounced stability effect. Thus, the two C8α and β variants not only show familial segregation but are also predicted to impact the formation, stability, or function of the heterotrimeric C8 complex.

### In-vitro analysis of the C8 triplex confirms the potential impact of variants on protein interaction

To investigate the effect of identified variants on the assembly of the C8 triplex, we co-transfected all three C8 subunits, with and without variants, in HEK293 cells (Figure S2). All constructs could form a C8 complex, as indicated by co-immunoprecipitation (Co-IP) experiments (Figure 3C). As predicted by stability modeling, the introduction of the C8 variants increased the immunoprecipitation of C8 subunits with C8α or C8β antibody (Figure 3D). Notably, the C8α variant displayed a higher affinity to C8G as shown by the higher amount in co-immunoprecipitation experiment (Figure 3E), confirming previous reports of C8A R444 facilitating the interaction with C8G.

## Discussion

GWAS using common variants have identified genetic loci of interest for many complex traits, but rare variants in familial cases can help ascertain causal genes and provide clues to molecular mechanisms. Clustering of AMD in familial structure, mimicking an autosomal dominant mode of inheritance, is strongly indicative of genetic variants having a large effect size. In concordance, we uncovered ultra-rare missense variants in *C8A* and *C8B* genes segregating in 4 families, and no other reported AMD-associated common or rare variant^6^^,^^9^ showed segregation with AMD phenotype. Furthermore, the rare variants in both α or β C8 subunits could impact C8 heterotrimer formation and/or stability, likely altering MAC-mediated immune response. In addition, a recent study identified C8G as an inhibitor of neuroinflammation,^12^ our results show that C8 variants could affect the C8G binding, and thus potentially prevent its role as an inhibitor and exacerbate the inflammatory process in AMD.

Three C8 subunits (α, β, and γ encoded by *C8A*, *C8B*, and *C8G* genes, respectively) form the terminal MAC together with C5b, C6, C7, and C9.^13^ A rare variant in C9, previously implicated in AMD, is shown to enhance MAC polymerization.^14^ Nonsense mutations in C8 can cause C8 deficiency,^15^ but their impact on vision has not been assessed.

The three complement pathways (classical, lectin and alternative) converge on C5b-guided sequential assembly of C6-C9 resulting in the formation of MAC, and deposition or activation of MAC can initiate divergent signaling pathways triggering an immune response and cellular defense mechanisms.^7^^,^^16^ Together with the reported rare variant in *C9*,^14^ our study further strengthens the hypothesis of dysregulated terminal MAC as a key contributor to AMD pathology and suggests that rare variants in C8 genes could also affect the complex formation or its stability.

Molecular diagnosis is not a part of routine AMD screening, and aside from the two strongly associated common genetic variants (ClinVar: rs1061170 in CFH and ClinVar: rs10490924 in ARMS2/HTRA1), patients are generally not tested for any of the other common or rare variants that are reported in the literature. Most clinical studies have also focused on the correlation of only these two variants with divergent disease phenotypes. Notably, common variants in distinct complement genes have been linked to both protective and deleterious effects on disease progression, in addition to stage-specific (and at times conflicting) effects.^17^ Except for C9, all reported variants in the complement cascade are clustered in the early steps of the pathway, leading to a concentrated effort to modulate or augment these steps for the development of therapies. Of the 21 targets specifically directed to the complement cascade, only one trial is focusing on the MAC, using an AAV to produce CD59, which prevents the binding of C9 and disrupts the assembly of the MAC (ClinicalTrials.gov: NCT04358471).^18^ However, their success in clinical trials has been limited at best.^19^

**Figure 4:**
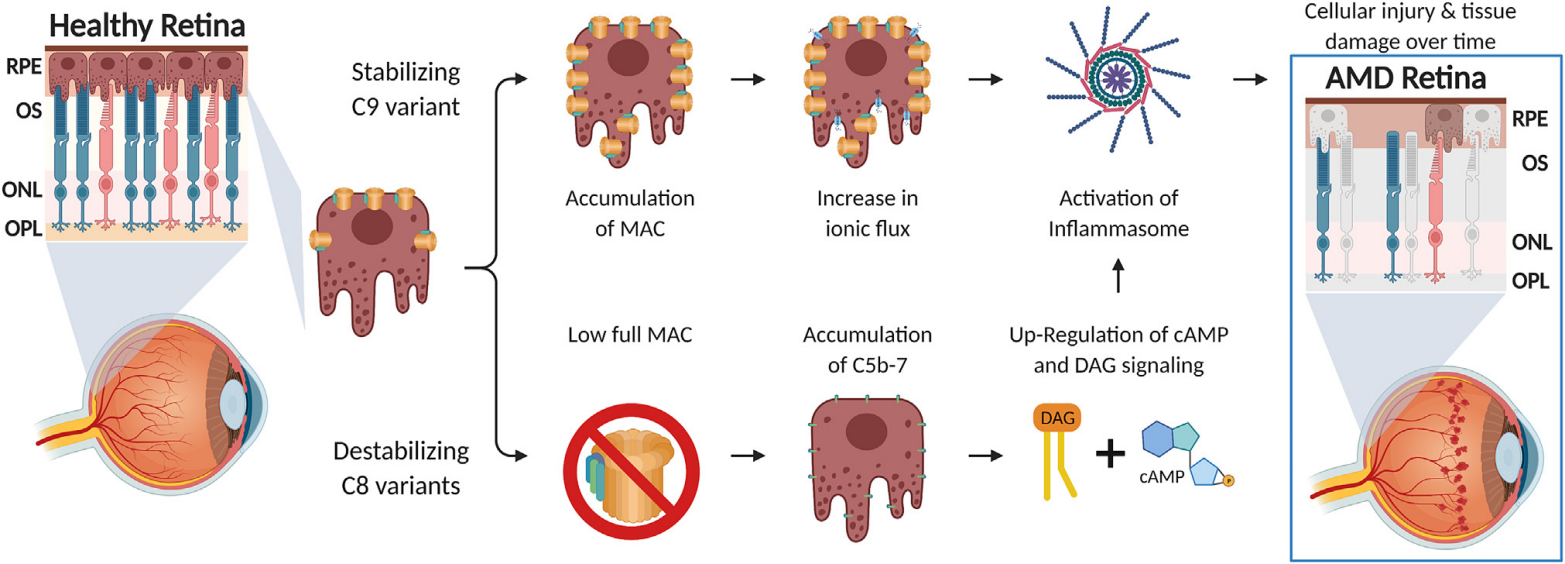
A schematic of the proposed model for the mechanism of MAC-associated AMD pathology

The concept of “inflammaging” was introduced to provide a framework for many age-related conditions, including AMD,^20^^,^^21^ with chronic low levels of inflammation being a key contributor. Inflammasome activation has been reported in RPE from AMD patients as well as in ARPE-19 cells.^22^^,^^23^ Furthermore, multiple studies have shown that MAC dysregulation can lead to inflammasome activation.^24^^,^^25^^,^^26^^,^^27^^,^^28^ Notably, inflammasome activation can drive inflammation in *C9*^*−/−*^ mice lacking the ability to form MAC.^24^ These studies, together with our data, indicate a complex interplay between AMD, MAC and the inflammasome. In Figure 4, we propose a model to explain the role of MAC in AMD. Terminal MAC can therefore be mentioned as a target of therapies for age-related diseases such as AMD, where “inflammaging” might be a contributor to pathology.

### Limitation of the study

Our genetic analysis is based on exome capture, which is limited in the coverage of the genome to the (mostly) coding regions that are covered by the probes provided. This might lead to incomplete coverage of some genes or regions, particularly in the regulatory or deep-intronic regions. WES is also prone to uneven coverage because of differences in binding of the probes, leading to improper detection of variants.

The effect of variants is deduced by computational analysis or in HEK293 cells. Further investigations are needed with relevant target tissues or cells and using *in vivo* model systems to examine the contribution of C8 and MAC in AMD pathology.

## STAR★Methods

### Key resources table

**Table:** 

REAGENT or RESOURCE	SOURCE	IDENTIFIER
**Antibodies**		

C8A	Abcam	Cat# ab273626
C8B	Abcam	Cat# ab278045
C8G	Abcam	Cat# ab181182
C7	LSBio	Cat# LS-B10578
C9	R&D Systems	Cat# MAB8126
Anti-Rb IgG HRP	Jackson ImmunoResearch	Cat# 711-035-152; RRID:AB_10015282
EasyBlot anti-Rabbit IgG HRP	GeneTex	Cat# GTX221666-01; RRID:AB_10620421
Anti-Ms HRP	Jackson ImmunoResearch	Cat# 715-035-150; RRID:AB_2340770
Anti-Gt HRP	Millipore	Cat# AP180P; RRID:AB_92573
Mouse anti-beta Actin	Millipore/Sigma	Cat# A5316; RRID:AB_476743

**Biological samples**		

Human RPE	National Disease Research Interchange (NDRI); RRID:SCR_000550	N/A
Patients DNA samples	This paper	N/A

**Critical commercial assays**		

Q5® Site-Directed Mutagenesis Kit	New England Biolabs	E0554S

**Deposited data**		

Whole exome and Sanger sequencingdata was deposited to NCBI SRA.	This Paper	PRJNA805222
ExAC carrier rates were obtained from gnomAD browser	gnomAD	RRID:SCR_014964, https://gnomad.broadinstitute.org/

**Experimental models: Cell lines**		

HEK293T	ATCC (Manassas, Virginia)	Cat# CRL-3216; RRID:CVCL_0063

**Oligonucleotides**		

C8A and C8B Plasmid primers	IDT	See constructs section under method details
C8A genotyping primers	ThermoFisher	Hs00399162_CE (A15629,A15630)
C8B genotyping primers	ThermoFisher	Hs00437310_CE (A15629,A15630)

**Recombinant DNA**		

pCMV3-C8A-GFP	LSBio (Seattle, WA)	LS-N187463
pCMV3-C8B-GFP	LSBio (Seattle, WA)	LS-N31526
pCMV3-C8G-DDK	LSBio (Seattle, WA)	LS-N60238

**Software and algorithms**		

FastQC (Version 0.11.9)	Babraham Bioinformatics	https://www.bioinformatics.babraham.ac.uk/projects/fastqc/
Trimmomatic (Version 0.36)	Bolger et al.,^29^	http://www.usadellab.org/cms/?page = trimmomatic
BWA (Version 0.7.17)	Li and Durbin,^30^	https://sourceforge.net/projects/bio-bwa/files/
Picard (Version 2.17.11)	Broad Institute	https://broadinstitute.github.io/picard/
GATK (Version 3.8-1)	Broad Institute	https://github.com/broadgsa/gatk
ANNOVAR	Wang et al.,^31^	https://annovar.openbioinformatics.org/en/latest/
PLINK (Version 1.9)	https://www.cog-genomics.org/plink/	https://www.cog-genomics.org/plink/
R v4.0.3	https://cran.r-project.org/	https://cran.r-project.org/
SWISS-MODEL server	Swiss Institute of Bioinformatics Biozentrum, University of Basel	https://swissmodel.expasy.org
Yasara	http://yasara.org	http://yasara.org
PyMol	PyMOL by Schrödinger	https://pymol.org/2/
FoldX	Center for Genomic Regulation	https://foldxsuite.crg.eu
Image Lab	BioRad	https://www.bio-rad.com/en-us/product/image-lab-software?ID=KRE6P5E8Z

**Other**		

AREDS1 and AREDS2 genotyping data	1417-1436. https://doi.org/10.1001/archopht.119.10.1417, https://doi.org/10.1001/jamaophthalmol.2013.4412	1417-1436. https://doi.org/10.1001/archopht.119.10.1417, https://doi.org/10.1001/jamaophthalmol.2013.4412
BioRender	https://biorender.com/	https://biorender.com/
Biowulf cluster	http://hpc.nih.gov	http://hpc.nih.gov

### Resource availability

#### Lead contact

Further information and requests for resources should be directed to and will be fulfilled by the lead contact, Anand Swaroop (swaroopa@nei.nih.gov) and Michael L. Klein (kleinm@ohsu.edu).

#### Materials availability

This study did not generate new unique reagents.

### Experimental model and subject details

#### Study cohort

This research was approved and conducted in accordance with the Oregon Health & Science University Institutional Review Board. This cohort is comprised of 152 families recruited in the Pacific Northwest United States, with 1287 ascertained family members phenotyped for AMD. Patients seen at the Casey Eye Institute (Portland, OR, USA) who were identified as having a family history of AMD were invited to participate in genetic research and to assist us in reaching out to other members of their family. All subjects enrolled in the study agreed to participate after informed consent. Participants were asked to provide their medical history of AMD including fundus photographs, provide a blood sample, answer a questionnaire, describe known family history of AMD, and agree to follow up communications. The questionnaire included questions regarding demographics, ocular disease history, cardiovascular disease history, diabetes history, smoking habits, medications, and supplements. Over the years, many participants were re-contacted for follow up information and to obtain additional fundus photographs. All fundus images were carefully graded using a modification of the AREDS scale.^32^ Each eye was graded as follows based on drusen size and/or evidence of GA or CNV, and the phenotype category for the worse eye was used for each individual.

**Table:** 

Grade	Fundus findings	Phenotype category
1	None or drusen <63 μm	None or small drusen
2	Drusen 63-124 μm	Medium drusen
3	Drusen 125-249 μm, or >249 μm	Large or very large drusen
4	Either GA or CNV in one eye	Advanced AMD
5	Either GA or CNV in both eyes	Advanced AMD

Whole blood was collected in anti-coagulation tubes and processed for extraction of genomic DNA using standard methods. Genomic DNA stocks were stored at −80°C. All phenotype, self-reported questionnaire results, and family relationship data were maintained in a Progeny database (Progeny Genetics, Delray Beach, FL, USA).

#### Cell culture

HEK293T were originally obtained from the AmericanType Culture Collection (ATCC, Manassas, Virginia) and were cultured in Dulbecco’s Modified Eagle’s Medium (Invitrogen, 12800-017) supplemented with 10% fetal bovine serum according to standard protocols. Cells were grown at 37°C in humidified air containing 5% CO_2_ and subcultured every 2-3 days by trypsinization (Invitrogen, 12604-02). Cells were not tested for mycoplasma and were not authenticated.

### Method details

#### Sanger sequencing

Genomic DNA (40 ng) was subjected to PCR to amplify the region around each variant in 20 μL reactions using AccuPower PCR Pre-Mix (Bioneer) according to manufacturer’s instructions. The primers (1 μM of a forward and reverse primer per PCR reaction, ThermoFisher Catalog A15629/A15630) and PCR reaction conditions are detailed in the table below. An aliquot of each PCR reaction was visualized on a 2% agarose gene to ensure proper amplification. Products were cleaned up with QIAquick PCR purification kit (Qiagen) according to manufacturer’s instructions. The purified products were quantified and sequencing reactions containing 9 ng PCR product with 2.13 μM forward or reverse primer were subjected to Sanger sequencing in the Vollum Institute, DNA Sequencing Core Facility (OHSU, Portland, OR).

**Table:** 

	C8A	C8B
ThermoFisher Primer ID	Hs00399162_CE	Hs00437310_CE
Forward primer	ACTTTCTTCCAAATCTCATTAGTGGG	GGACATAGTTGGCCTAGAAGTTGGG
Reverse primer	AACTTCTGGGATGAGCAGTGTAAA	GGCTGAAAGGGAAGCTGGACC
CGRCh38 location	Chr.1: 56907865-56908364	Chr.1: 56943571-56944035
Amplicon Length (bp)	500	465
PCR denaturation	94°C, 30 s.	94°C, 30 s.
PCR annealing	59.5°C, 30 s.	63.8°C, 30 s.
PCR extension	72.0°C, 60 s.	72.0°C, 60 s.
Number of PCR cycles	35	35

#### Whole exome sequencing (WES)

Genomic DNA was extracted from peripheral blood samples using standard methods and stored at −80°C until further use. Prior to sequencing gDNA concentration was determined using nanodrop (at OHSU) and QuantiFluor dsDNA System by Promega (at NEI), according to the manufacturer’s instructions. WES DNA libraries (from DNA samples of individuals selected for WES) and sequencing was done in the Cancer Genomics Research Laboratory (CGR), Leidos Biomedical Research, Frederick National Laboratory for Cancer Research, Frederick, MD, USA using standard protocols. Pair-ended 149bp sequencing was performed on NovaSeq 6000 (Illumina, Inc., San Diego, CA).

#### Bioinformatics analysis

Raw reads were obtained in FASTQ format from Illumina NovaSeq 6000 sequencing platform. Reads were assessed for quality control using FastQC (Version 0.11.9) (https://www.bioinformatics.babraham.ac.uk/projects/fastqc/). Adapter sequences were removed using Trimmomatic (Version 0.36)^29^ and clean reads were aligned against human reference genome GRCh38 using BWA (Version 0.7.17).^30^ Binary alignment map (BAM) files were further processed to mark duplicate reads with Picard (Version 2.17.11) (https://broadinstitute.github.io/picard/). BAM files with duplicate reads marked were subjected to local realignment, base quality recalibration, variant recalibration, and variant calling using GATK (Version 3.8-1).^33^ Variant annotation was carried out using ANNOVAR.^31^ Sex check and identity by descent (IBD) analysis for all the samples was carried out using PLINK (Version 1.9).^34^ This work used the computational resources of the NIH HPC Biowulf cluster (http://hpc.nih.gov).

#### Variant filtering

Filtration of WES variants was carried out and variants in category “exonic” and “splicing” were considered for further analysis with CADD score ≥15, CADD score = “.”. List of variants was retained with MAF ≤1% and MAF = “.” as per ExAC_all.

We then applied a segregation filter. Variants segregating in all affected individuals or all affected individuals but one within a family were retained. Finally, the variant level data were collapsed into gene-level data by combining all variants observed in each gene across different families.

#### Constructs

pCMV3-C8A-GFP (LS-N187463), pCMV3-C8B-GFP (LS-N31526), and pCMV3-C8G-DDK (LS-N60238) expression vectors were all purchased from LSBio (Seattle, WA). AMD-associated variants were introduced into the corresponding C8A and C8B ORFs using the Q5 Site-Directed Mutagenesis Kit (New England Biolabs). Original ORF sequence and mutated ORF sequence were verified by Sanger sequencing. The mutagenesis and sequencing primers used are listed.

**Table:** 

Oligo Name	Oligo Sequence
C8A_mut_F	CATTACATACCATTCCTGGGGGAG
C8A_mut_R	GTGCTCCTGTTCTGTGCCAAGCCA
C8A-nt417-F	CTGCAGCCAGTATGAACCAA
C8A-nt1080-R	TGCTTTGTCAATCACCAGGA
C8A-nt1011-F	TGGCACCCATTACATCACAT
C8A-nt1753-R	AGCAAGCCTGCGTCTGTACT
C8B_mut_F	GCCAAAAATTATTTTAAAATTGGTGGTGCC
C8B_mut_R	ACAGGCATGGACGTTGTTAAGAG
C8B_nt420_F	CAATGGGGACAATGACTGTG
C8B_nt1198_R	CAGACACACCCAGACTGACG
C8B_nt1157_F	GTGGTGCCATTGAAGAGGTC
C8B_nt1773_R	GGAGCAGTCAAGTGTTTCTGAAG

#### Cell transfections

Before transfection 2 × 10^5^ HEK293 cells were seeded into individual wells of 6 well plates. After a 48 h incubation in growth medium, transfections were performed using lipofectamine 2000 (Invitrogen) according to the manufacturer’s protocol. The ratio Lipofectamine 2000/DNA was 3:1. After transfection, the cells were incubated at 37°C in a humidified incubator with 5% CO_2_ for 24 h. Each transfection was carried out in triplicates and repeated 3 times. Cells were co-transfected with the following combination of plasmids (500 ng each): (1) C8A-GFP, C8B-GFP, and C8G-FLAG, (2) C8A-Variant-GFP, C8B-GFP and C8G-FLAG, and (3) C8A-GFP, C8B-Variant-GFP, and C8G-FLAG.

#### Protein extraction and Co-Immunoprecipitation

Cells were rinsed in Dulbecco’s phosphate-buffered saline (DPBS), harvested, and lysed in Pierce IP Lysis Buffer (Thermo Scientific) supplemented with complete protease and PhosSTOP phosphatase inhibitor cocktails (Roche) on ice for 30 min. BCA protein assays were performed to quantitate protein according to instructions supplied by the manufacturer (Pierce). Protein lysates (450 μg) were incubated with 1 μg of rabbit monoclonal antibodies against C8A or C8B at 4°C overnight. Rabbit IgG (1 μg, Jackson ImmunoResearch Laboratories) was used as a negative control. The next day, Dynabeads Protein A magnetic beads (Invitrogen) were added, and the mixtures were gently mixed at 4°C for 4.5 h. Thereafter, the beads were isolated and washed with wash buffer (phosphate buffered saline (pH 7.4) with 0.02% Tween 20 in presence of protease and phosphatase inhibitors cocktails), and the protein level of C8A, C8A variant, C8B, C8B variant and C8G were analyzed by western blot.

#### Immunoblot analysis of cultured cells

Total protein lysates or IP complexes were solubilized in 4× Laemmli buffer supplemented with β-mercaptoethanol and incubated for 5minat 95°C. Samples were loaded and resolved using precast 10% sodium dodecyl sulfate-polyacrylamide gel electrophoresis (SDS-PAGE) gels from Bio-Rad (Mini PROTEAN TGX Gel) and electro-transferred to PDVF membranes using Bio-Rad Trans-Blot Turbo Transfer System (Bio-Rad). Standard immunoblot procedures were followed using primary antibodies against human C8A, C8B and C8G raised in rabbit (Abcam). Briefly, the blots were blocked with 5% EasyBlocker (GeneTex) dissolved in TBST buffer and incubated with primary antibodies at 1:1,000 dilution overnight at 4°C. The following day, the blots were washed and incubated with HRP-conjugated secondary antibodies (EasyBlot anti-rabbit IgG (1:1,000) or donkey anti-rabbit IgG (1:10,000)) at room temperature for 1 h. SuperSignal West Pico PLUS Chemiluminescent Substrate (Thermo Scientific) was used for signal detection. All membranes were imaged using the ChemiDoc Imaging System (Bio-Rad).

#### RPE protein extraction and immunoblot analysis

A single postmortem human eye were obtained from the National Disease Research Interchange (NDRI, Philadelphia, PA, US). RPE was dissected out of the eye and kept at −80°C until further use. 140 mg of tissue was defrosted on ice and processed for protein extraction using RIPA buffer (50 mM Tris-HCl pH7.4, 150 mM NaCl, 1% NP-40, 1 mM EDTA, 1 mM EGTA, 0.5% Sodium Deoxycholate and 0.1% SDS) supplemented with protease and phosphatase inhibitors (Roche) and two cycles of sonication (Amp 10%, 3 s). Protein concentration was determined using Pierce BCA Protein Assay Kit (Thermo Scientific). 20 μg of protein was incubated for 5minat 95°C with 4x Laemmli Sample Buffer (1610747, Bio-Rad). The protein solution was loaded and resolved using precast 10% sodium dodecyl sulfate-polyacrylamide gel electrophoresis (SDS-PAGE) gels from Bio-Rad (Mini PROTEAN TGX Gel) and electro-transferred to PDVF membranes using BioRad Trans-Blot Turbo Transfer System (Bio-Rad). The blots were blocked with 5% milk powder in TBST and incubated with primary antibody; O/N at 4°C, followed by three washes with TBST and then 1-h incubation at RT with species-appropriate secondary antibody conjugated with horseradish peroxidase (HRP). SuperSignal West Pico PLUS Chemiluminescent Substrate (Thermo Scientific) was used for signal detection. All membranes were imaged using The ChemiDoc Imaging System (Bio-Rad).

#### Antibodies used in this study

**Table:** 

	Antibody	Company	Catalog number	Method used
1	C8A	Abcam	ab273626	WB (1:1,000), IP (1 μg)
2	C8B	Abcam	ab278045	WB (1:1,000), IP (1 μg)
3	C8G	Abcam	ab181182	WB (1:1,000)
4	C7	LSBio	LS-B10578	WB (1:1,000)
5	C9	R&D Systems	MAB8126	WB (1:500)
6	Anti-Rb IgG HRP	Jackson ImmunoResearch	711-035-152	WB (1:10,000)
7	EasyBlot anti-Rabbit IgG HRP	GeneTex	GTX221666-01	WB (1:1,000)
8	Anti-Ms HRP	Jackson ImmunoResearch	715-035-150	WB (1:10,000)
9	Anti-Gt HRP	Millipore	AP180P	WB (1:10,000)
10	Mouse anti-beta Actin	Millipore/Sigma	A5316	WB (1:1,000)

#### Molecular modeling

Amino acid sequences of human complement component C8 alpha (UniProtKB: CO8A, acc. #P07357), beta (UniProkKB: CO8B, acc. #P07358), and gamma (UniProtKB: CO8G, acc. #P07360) chains were obtained from the UniProt database (https://uniprot.org/uniprot/). The model of the trimeric hetero-complex C8 was built using oligomeric protein modeling options in a fully automated protein structure homology modeling SWISS-MODEL server (https://swissmodel.expasy.org)^35^^,^^36^ and a molecular-graphics,-modeling and-simulation program Yasara (http://yasara.org).^37^^,^^38^ The following files from the RCSB PDB database (https://www.rcsb.org) 2rd7, 3ojy, and 2gos were used as structural templates to build the homology model of the C8 trimer. PyMol (https://pymol.org/2/) was used for visualization (variants introduced using the mutagenesis option in the wizard) and hydrogen bond interactions. Mutant variants R444H (CO8A subunit) and D382Y (CO8B subunit) were generated by the ‘edit: swap’ procedure and equilibrated using 500 ns molecular dynamics in water in Yasara. Protein stability was evaluated using the FoldX plugin in Yasara.^39^ Before protein stability calculations, trimeric molecules C8, isolated alpha and beta subunits, and corresponding mutant variants were repaired by FoldX (https://foldxsuite.crg). Changes in protein stability were calculated as the following:

ΔΔG = ΔG(mutant) - ΔG(wild type).

Here ΔG(mutant) and ΔG(wild type) are protein stabilities for mutant variant and wild type protein respectively.

Structural model files (PDB format) are included as separate files (Data S2).

### Quantification and statistical analysis

#### AREDS1 and AREDS2 analysis

Variants called from whole-genome sequencing of the AREDS and AREDS2 cohorts were filtered to only C8A rs143908758 and C8B rs139498867. Only AMD cases were kept, of any subtype (N = 2451). A custom R script (R version 4.0.3) processed VCF data and counted the number of heterozygous carriers, homozygous carriers, and non-carriers. The sum of all carriers with AMD was compared to the number of carriers in the ExAC exome sequencing cohort (N = 60706). ExAC carrier rates were obtained from gnomAD browser.^40^ Chi-squared tests were run to approximate the expected carrier and noncarrier counts for AMD and ExAC samples. Since the number of carriers was small, Fisher’s Exact tests were run. P-values from Fisher’s Exact and carrier counts were reported. R packages used included data.Table 1.14.2, ggplot2 3.3.5, and base stats 4.0.3. Samtools and bcftools from htslib 1.13 were used to manipulate VCFs.^41^ This work used the computational resources of the NIH HPC Biowulf cluster (http://hpc.nih.gov).

#### IP signal quantification

Raw images of IP membranes were analyzed using Image Lab software (BioRad). Signal intensity was determined by selecting the appropriate bands on each membrane using the lanes and bands function in the software. Background level subtraction was done as recommended by the manufacturer tutorial. Adjusted signal intensity (found in supplementary Excel file 1) was exported to an Excel file for further analysis. To enable comparison the intensity of the protein bands in each transfection and IP conditions was quantified as previously described in the STAR protocol.^42^ Briefly, the intensity was first normalized to the amount of protein in the input control (indicating the total amount of protein generated in the cells) and then divided by the quantity in the WT conditions, for ease of visualization. WT levels are always indicated by 1, increase in the amount of protein will give a ratio >1, decrease in the amount of protein will give a ratio <1. Wilcoxon test was performed to assess statistical significance.

## Data Availability

• All data reported in this paper will be shared by the lead contact upon request and is available at NCBI SRA bioproject PRJNA805222. • Any additional information required to reanalyze the data reported in this paper is available from the lead contact upon request. • Whole exome and Sanger sequencingdata were deposited to the NCBI SRA under bioproject PRJNA805222.

## Supplemental information

Document S1. Figures S1 and S2 and Table S2 and S3

Data S1. Table S1 – Clinical data summary, related to Figure 1 A table summarizing all the clinical and genetic information about the families recruited to the study. Medical history is based on self-reporting. AAMD, Advanced age-related macular degeneration; GA, Geographic atrophy; NV, Neovascularization; DPED, Drusenoid pigment epithelial detachment; VA, Visual acuity; CF, Counting fingers; HM, Hand motion; LP, Light perception; ERM, Epiretinal membrane; RPE, Retinal pigmented epithelium; WES, Whole exome sequencing; CHF, Congestive heart failure; BMI, Body mass index; OD, Right eye; OS, Left eye; OU, both eyes.

Data S2. Computational model of C8 structure, related to Figure 3
