# Supplementary material for: Ultra-rare complement factor 8 coding variants in families with age-related macular degeneration
Source: iScience. 2023 Apr 3;26(4):106417. doi: 10.1016/j.isci.2023.106417 (PMC10156737; doi:10.1016/j.isci.2023.106417)
Supplement: Document S1. Figures S1 and S2 and Table S2 and S3 [file mmc1.pdf]

iScience, Volume *26*

Supplemental information

Ultra-rare complement factor 8 coding variants

in families with age-related macular degeneration

Lina Zelinger, Tammy M. Martin, Jayshree Advani, Laura Campello, Milton A. English, Alan Kwong, Claire Weber, Jennifer Maykoski, Yuri V. Sergeev, Robert Fariss, Emily Y. Chew, Michael L. Klein, and Anand Swaroop

Supplemental item titles and legends

Table S2 – Variants in C8A and C8B identified in the study, Related to Figure 1 and 2.

	**Variant 1**	**Variant 2**
**Gene**	*C8A*	*C8B*
**Ref Transcript**	NM_000562	NM_000066
**Change DNA**	c.G1331A	c.G1144T
**Change Protein**	p.R444H	p.D382Y
**Genomic Position (GRCh38)**	chr1:56908064	chr1:56943786
**RS_ID**	rs143908758	rs139498867
**Ref Allele**	G	G
**Alt Allele**	A	T
**ExAC_ALL**	0.004	0.0042
**gnomAD**	0.003971	0.006335
**SIFT**	deleterious	deleterious
**Polyphen**	probably_damaging	possibly_damaging
**CADD Score**	32	28.2
**# of families identified**	1	3

Table S3 – Protein stability calculation by FoldX, Related to Figure 3.

	**C8 complex**		**Individual subunit**	
**Subunit protein**	**ΔG kcal/mol**	**ΔΔG kcal/mol**	**ΔG,kcal/mol**	**ΔΔG, kcal/mol**
C8A, reference sequence	495.5	-	233.84	-
C8A, R444H substitution	462.3	-33.2	231.51	-2.34
C8B, reference sequence	495.5	-	230.05	-
C8B, D382Y substitution	418.6	-43.7	193.05	-37

Supplementary figures and legends

Figure S1 – Pathogenicity prediction and DNA level conservation of identified variants in C8A and C8B genes, Related to Figure 2.
Pathogenicity predictions are taken from gnomAD server from Broad Institute (https://gnomad.broadinstitute.org/).
DNA level conservation is from 100 vertebrate track (PhyloP) in UCSC genome browser.

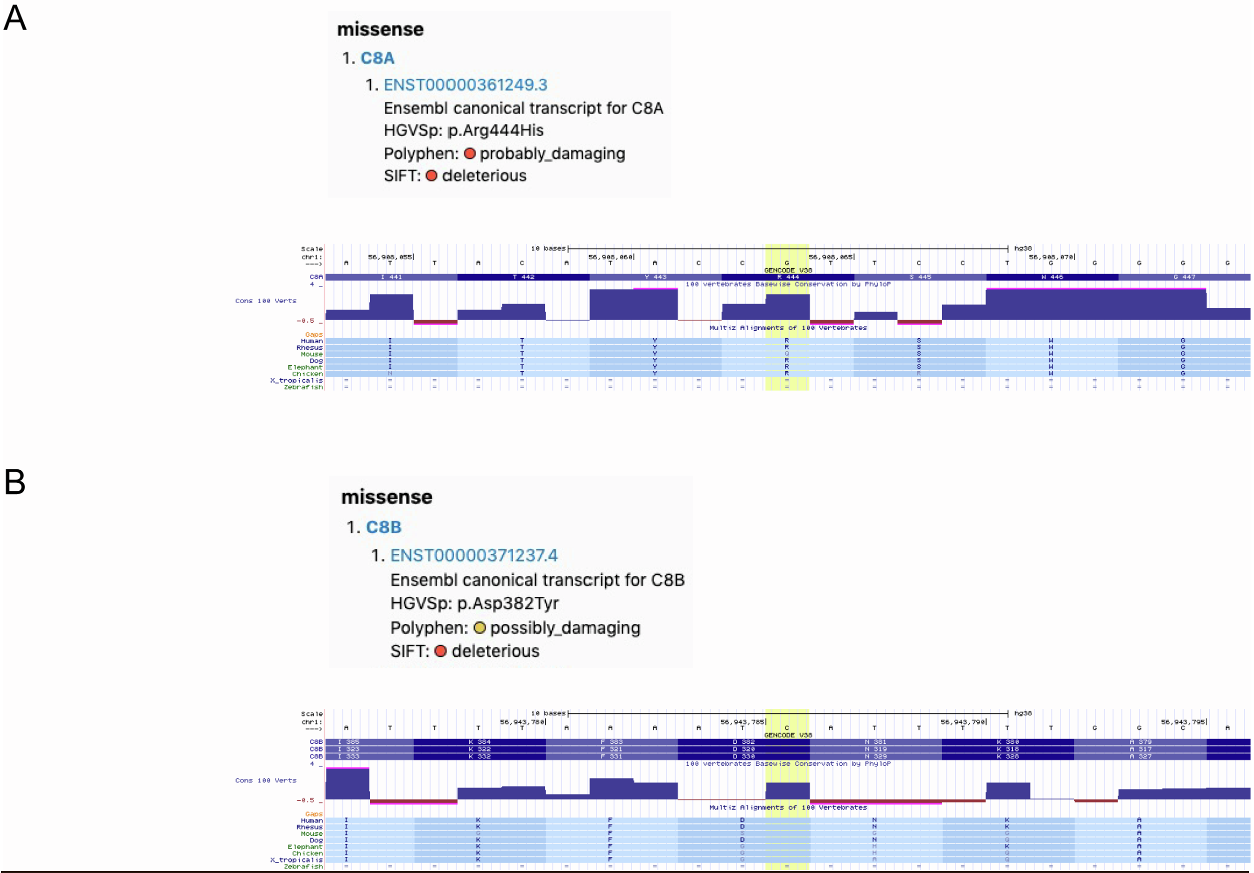

Figure S2 – Effect of variants on C8 complex subunits interaction, Related to Figure 3.

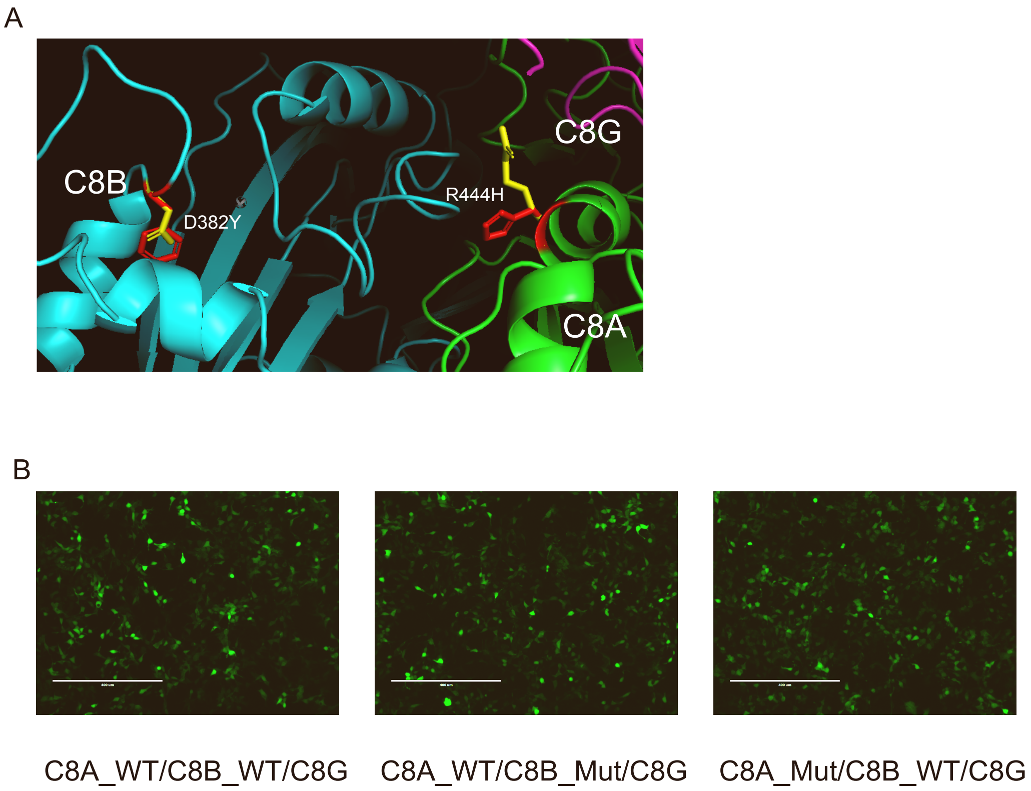

A. Snapshot of the C8 complex structure, showing both variants in red and WT residues in yellow. B. Representative images of transfection efficiency of different plasmid combinations in HEK293 cells. WT: wild type sequence; Mut: ultra-rare sequence variant.
