# Supplementary material for: Ultra-rare complement factor 8 coding variants in families with age-related macular degeneration
Source: iScience. 2023 Apr 3;26(4):106417. doi: 10.1016/j.isci.2023.106417 (PMC10156737; doi:10.1016/j.isci.2023.106417)
Supplement: Data S2. Computational model of C8 structure, related to Figure 3 [file mmc3.zip › C8_homology.docx]

Supplemental Information

C8-wt-500ps.pdb C8 homology model refined using 500 ps molecular dynamics

C8-D302Y-500ps.pdb Homology model of D302Y mutant variant refined using 500 ps molecular dynamics

C8-R444H-500ps.pdb Homology model of R444H mutant variant refined using 500 ps molecular dynamics
